# Miscarriage Tissue Research: Still in Its Infancy

**DOI:** 10.3390/life16010128

**Published:** 2026-01-14

**Authors:** Rosa E. Lagerwerf, Laura Kox, Melek Rousian, Bernadette S. De Bakker, Yousif Dawood

**Affiliations:** 1 Department of Obstetrics and Gynaecology, Amsterdam UMC, University of Amsterdam, Meibergdreef 9, 1105 AZ Amsterdam, The Netherlands; r.e.lagerwerf@students.uu.nl; 2 Department of Radiology, Leiden University Medical Center, Albinusdreef 2, 2333 ZG Leiden, The Netherlands; l.s.kox@amsterdamumc.nl; 3 Department of Obstetrics and Gynecology, University Medical Centre Rotterdam, Erasmus MC—Sophia Children’s Hospital, Dr. Molewaterplein 40, 3015 GD Rotterdam, The Netherlands; m.rousian@erasmusmc.nl; 4 Amsterdam Reproduction and Development Research Institute, 1105 AZ Amsterdam, The Netherlands; 5 Department of Paediatric Surgery, University Medical Centre Rotterdam, Erasmus MC—Sophia Children’s Hospital, Dr. Molewaterplein 40, 3015 GD Rotterdam, The Netherlands; 6 Department of Medical Biology, Amsterdam UMC, University of Amsterdam, Meibergdreef 9, 1105 AZ Amsterdam, The Netherlands

**Keywords:** miscarriage, pregnancy loss, genetic analysis, micro-CT, post-mortem imaging, karyotyping

## Abstract

Each year, around 23 million miscarriages occur worldwide, which have a substantial emotional impact on parents, and impose significant societal costs. While medical care accounts for most expenses, work productivity loss contributes significantly. Addressing underlying causes of miscarriage could improve parents’ mental health and potentially their economic impact. In most countries, investigations into miscarriage causes are only recommended after recurrent cases, focusing mainly on maternal factors. Fetal and placental tissue are rarely examined, as current guidelines do not advise routine genetic analyses of pregnancy tissue, because the impact of further clinical decision making and individual prognosis is unclear. However, this leaves over 90% of all miscarriage cases unexplained and highlights the need for alternative methods. We therefore conducted a narrative review on genetic analysis, autopsy, and imaging of products of conception (POC). Karyotyping, QF-PCR, SNP array, and aCGH were reviewed in different research settings, with QF-PCR being the most cost-effective, while obtaining the highest technical success rate. Karyotyping, historically being considered the gold standard for POC examination, was the least promising. Post-mortem imaging techniques including post-mortem ultrasound (PMUS), ultra-high-field magnetic resonance imaging (UHF-MRI), and microfocus computed tomography (micro-CT) show promising diagnostic capabilities in miscarriages, with micro-CT achieving the highest cost-effective performance. In conclusion, current guidelines do not recommend diagnostic testing for most cases, leaving the majority unexplained. Although genetic and imaging techniques show promising diagnostic potential, they should not yet be implemented in routine clinical care and require thorough evaluation within research settings—assessing not only diagnostic and psychosocial outcomes but also economic implications.

## 1. Introduction

Approximately 23 million miscarriages occur globally each year, with an estimate of 10–20% of confirmed pregnancies ending in miscarriage [1,2,3,4,5]. Expanding our knowledge of miscarriages, including their etiology, is crucial in preventing them in the future. This might reduce infertility and potentially boost birth rates, alleviating future social and economic pressures on our society. This issue has gained urgency as the global birth rate has significantly dropped over the last decades to 1.5 children per woman, falling far below the 2.1 needed for generational replacement [6]. According to the Organization for Economic Cooperation and Development, this decline, partly attributed to a rise in infertility affecting roughly one in six adults worldwide, poses a substantial threat to the prosperity of future generations by creating significant social and economic challenges in sustaining our society [7]. The World Health Organization has therefore emphasized the urgent need for improved fertility care and research into reproductive outcomes [8]. Besides these demographic concerns, miscarriages also have a significant economic impact, with an estimated short-term economic burden in the United Kingdom alone of £ 471 million annually [1].

The European Society of Human Reproduction and Embryology (ESHRE) defines a miscarriage as the loss of a medically confirmed pregnancy before 24 weeks gestational age, with the majority occurring before 12 weeks [5]. The etiology is multifactorial and chromosomal abnormalities represent the most prevalent cause. In about 45% of miscarriages, a chromosomal abnormality could be identified, compared to less than 1% in livebirths without prenatal testing [9]. Beyond embryonic causes, defects in endometrial decidualization due to immune or stem cell dysfunction can also lead to miscarriage [10,11,12]. Demographic and lifestyle risk factors, including advanced maternal age, high or low BMI, smoking, alcohol, caffeine intake, and night shift work, show varying degrees of association, often with dose–response relationships [1,13,14]. Clinical contributors include uterine anomalies, endocrine disorders (e.g., thyroid dysfunction and anti-phospholipid syndrome), and infections, while environmental exposures—such as air pollution and pesticides—have also been linked to an increased risk [1,15,16,17].

The true impact of miscarriage extends beyond demographic and economic dimensions. It has major consequences for the intended parents and family members, including potential physical risks for the mother, such as severe hemorrhage and complications in future pregnancies [1,3], but also more hidden—psychological—consequences. These often go unnoticed by family, friends, and healthcare professionals. This effect is magnified in societies that regard miscarriages as insignificant or shameful, leading to parents concealing their difficulties [1]. Experiencing a miscarriage is associated with complicated grief, anxiety, trauma, depression, and suicide [1,18,19]. A large study, following 908 women, found that nine months after women had experienced a miscarriage, 18% suffered from post-traumatic stress, 17% from anxiety, and 6% from depression [20]. Although often overlooked, partners and family members are also impacted by the loss, with one study reporting a prevalence of 9% for complicated grief and 4% for postpartum depression in men four months after pregnancy loss [21]. For many parents, the psychological burden of miscarriage is exacerbated by uncertainty, as they frequently seek explanations for their loss, even if this knowledge does not prevent a future miscarriage [1,22].

Despite this strong desire for answers, the vast majority of miscarriages is never subject to further medical investigation. Around 11% of women trying to conceive will experience one miscarriage [1]. Recurrent pregnancy loss (RPL), typically defined as the occurrence of two or more miscarriages, affects approximately 2.6% of women, including about 0.7% who report three or more losses [1]. Despite this prevalence, only a small subset of couples—those with multiple consecutive losses—are eligible for further diagnostic evaluation in Europe and the United States [5,23]. This evaluation, which applies to an estimated 1–3% of couples, primarily focuses on maternal risk factors. Fetal and placental tissue are rarely examined, as current guidelines do not advice routine genetic analyses of pregnancy tissue, because the impact of further clinical decision making and the exact influence on prognosis for an individual patient is unclear [24]. However, this leaves over half of these recurrent cases unexplained and highlights the need for alternative methods [2,3,23,25,26,27]. Consequently, in over 90% of all miscarriages no explanation is found for the miscarriage. Perhaps most strikingly, under current guidelines, no additional fetal testing is offered in an estimated 99% of all miscarriages. As a result, parents are left with unanswered questions—and more importantly, without guidance for future pregnancies.

In recent years, significant and promising progress has been made through the analysis of miscarriage tissue, also known as products of conception (POC), using genetics, autopsy, and imaging techniques in research settings. By deepening our understanding of miscarriages and their underlying etiologies, we may in the future be able to offer better informed medical guidance and support to parents during this difficult time. Thus, the objective of this narrative review is to explore possible techniques for POC examination to determine the cause of a miscarriage, including genetic analyses and post-mortem autopsy or imaging while evaluating the benefits and drawbacks of each approach.

## 2. Search

We performed a targeted literature search in PubMed, Embase, and Google Scholar using combinations of keywords and controlled vocabulary (where available) related to miscarriage, products of conception, genetic testing, autopsy, and post-mortem imaging. Reference lists of included papers were then extensively screened and iteratively expanded using backward and forward snowballing to identify additional relevant studies. This work was conducted as a narrative review (not a systematic review) and therefore did not follow a pre-registered protocol or apply formal study selection and risk-of-bias procedures.

## 3. Genetic Analysis

POC is a collective term encompassing all tissues formed during pregnancy that originate from the conceptus. POC includes, but is not limited to, fetal tissue, chorionic villi, amniotic membrane and fluid, the umbilical cord, and the placenta. There is currently no standardized protocol regarding which specific tissue type should be used for genetic analysis. Some studies utilize the chorionic villi [28,29] while some used multiple different POC [30,31,32], making cross-study comparisons challenging. It is important to note that each of these tissues has a distinct embryological origin and may therefore yield different genetic results in cases of mosaicism. A well-known example is the commonly performed non-invasive prenatal testing (NIPT), which uses DNA from trophoblast cells and therefore can produce false-positive results due to chromosomal mosaicism present in the placenta, but absent in the fetus itself [33]. To obtain the most accurate representation of the pregnancy’s chromosomal status, it is advisable to sample multiple types of POC tissue, thereby encompassing the various embryonic lineages. As different studies utilize various tissue types under the umbrella of POC, all such tissues will be collectively referred to as POC for the purposes of this review.

Genetic testing of the POC can be performed using various techniques. These include, but are not limited to, karyotyping, Fluorescence In Situ Hybridization (FISH), array-based Comparative Genomic Hybridization (aCGH), Single Nucleotide Polymorphism (SNP) array, Quantitative Fluorescence Polymerase Chain Reaction (QF-PCR), next generation sequencing, whole genome sequencing, and Multiplex Ligation-Dependent Probe Amplification (MLPA) [34,35]. In this review, we focus on karyotyping, QF-PCR, SNP array, and aCGH, as these are the most commonly utilized methods in miscarriage research.

In at least half of the early miscarriages, chromosomal anomalies could be detected in the POC [36,37]. The most common genetic causes of first-trimester miscarriages are aneuploidies and polyploidies [34,38]. Most frequently detected chromosome abnormalities are a single trisomy of chromosomes 13, 15, 16, 18, 21, 22, monosomy X, and triploidy [30,31,34]. For a more detailed comparison, an overview of the prevalence of chromosomal anomaly types in abnormal samples was generated using three different studies (Table 1). Some abnormalities, such as a monosomy other than X or a double triploidy, are quite rare and are therefore scaled under ‘other’. The difference in prevalence of chromosomal abnormalities between the studies could be explained by the fact that these studies all examined a different population, used different POC types, and a range of detection techniques.

### 3.1. Karyotyping

The current historically considered gold standard for testing POC is karyotyping [34]. Karyotyping is a technique in which chromosomes are visualized by culturing fetal cells, inducing metaphase and staining them with G-banding technique after which they can be analyzed [28,39]. It can detect aneuploidy, polyploidy, large structural arrangements above 5 Mb, molar pregnancies, and, in some cases, mosaicism [28,39]. It can also recognize Robertsonian translocations, occurring in 0.5% of POCs, which is beyond the capabilities of some of the other techniques. Due to the low resolution, it cannot detect small structural rearrangements [40].

The technique is ideally performed on fresh tissue, since cell culture is required. This makes it less effective for miscarriages, with a success rate of only 60% in general [28]. When culture failure occurs, additional aCGH testing showed that 70% of these POC samples contained a chromosomal anomaly, which is higher than the average percentage of chromosomal anomalies in POCs, typically around 50% [32,36,37]. These findings imply that the technique’s reliance on cell culture may lead to a lower-than-expected technical success rate of detecting actual chromosomal anomalies in miscarriages. Furthermore, maternal cells can contaminate the fetal cell cultivation, occurring in an estimate of 29–58% of the 46,XX results [41]. Thus, it is advised that every 46,XX result is analyzed further with other detection methods to ensure no maternal cell contamination (MCC) has occurred [31]. The need for cell cultures makes karyotyping a lengthy, labor intensive, and expensive procedure, often taking up to 23 days to complete with an estimated cost ranging from € 800 to € 6000 [40,42,43]. However, karyotyping is a widely applied technique, and therefore highly accessible [44].

### 3.2. Quantitative Fluorescence Polymerase Chain Reaction

QF-PCR is a technique that uses fluorescent primers binding to short tandem repeats (STR) across a chromosome to quantify the amount of DNA present utilizing a capillary electrophoresis system. The quantification of DNA present corresponds to the number of copies of a chromosome, thereby indicating whether aneuploidy is present or not [34,45]. STR markers are used for chromosomes 13, 18, 21, X, and Y, and if desired, extra chromosomes such as 15, 16 and 22 may also be incorporated [30,45]. Since QF-PCR does not require cell culture, it is a rapid process with an estimated turnover time of one to three days [45,46]. Furthermore, QF-PCR is able to identify MCC, mosaicisms, triploidy, unbalanced rearrangements, and molar pregnancies; however, balanced structural rearrangements and tetraploidy are not detected [30,47,48]. The costs of QF-PCR were reported in one study conducted in South Africa, where the average cost was R1800, approximately €94, highlighting it as a relatively inexpensive and accessible alternative; however, this may not be directly translatable to other countries [45].

One study compared karyotyping with QF-PCR in fetal tissue. 63.5% of the 148 studied samples were successfully karyotyped while 94% could be successfully analyzed with QF-PCR. A corresponding diagnosis between the two techniques was identified in 81 out of 89 cases [48]. QF-PCR is often combined with other techniques using QF-PCR as the first step in analysis, because it is rapid and cost-effective, after which other techniques such as aCGH or MPLA are employed when QF-PCR yields a normal result [29,30]. One study compared QF-PCR combined with MPLA to karyotyping, showing that QF-PCR/MPLA had a higher success rate, lower turnover time, was less labor-intensive, and more affordable than conventional karyotyping [44]. Another study reported that QF-PCR successfully identified aneuploidy in 25% of cases, with a failure rate of 1.4%. QF-PCR samples with a normal result were then analyzed with aCGH after which an abnormality was detected in just 9.6% [30]. Combining QF-PCR with MPLA and comparing it to SNP array analysis showed that QF-PCR/MLPA achieved a comparable diagnostic yield of 59.6% similar to 60.3% with SNP array. Demonstrating that QF-PCR/MLPA provided a reliable, rapid, and cost-effective alternative to SNP arrays [29].

### 3.3. Chromosomal Microarray

Chromosomal microarray (CMA) is a collective term for tests using microarray probes, including SNP array analysis and aCGH. The probes are mounted on a chip and the patient’s DNA sample and a control DNA sample are added, after which the DNA fragments hybridize to the corresponding probes. A fluorescent signal is emitted which can be compared to the control DNA signal. An increase in the subject DNA suggests a gain in copy number, while a decrease indicates a loss at the genomic loci represented by the microarray probes. SNP array uses SNPs as its probes, while aCGH uses CNVs [49].

CMA has been proved useful for genetic analysis of POC. One large study including 8118 samples showed a success rate of 92.4% using CMA on fresh POCs. This study also examined POCs, which were formalin-fixed paraffin-embedded (FFPE) and showed an 86.4% success rate. This study used SNP array and aCGH as CMA techniques; however, a distinction between the two techniques in success rate was not made [50]. Another study compared SNP array, aCGH, and conventional karyotyping in sixty chorionic villi samples, collected from miscarriages treated with dilation and curettage. Each sample underwent all three tests, and the combined results determined whether it was classified as abnormal, which was the case for 78% of the POC samples. If a result was concordant with at least one other form of test, it was defined as a correct call. A correct call rate was calculated and resulted in being 85%, 93%, and 85% for karyotyping, SNP array, and aCGH, respectively. It is important to note that due to the clinical setting of this research, there was a short time interval between the miscarriage and collection of the tissue; therefore it was of higher quality and likely contributed to the high accuracy of the karyotyping results [40].

SNP array and aCGH have the ability to detect aneuploidy, polyploidy, and unbalanced rearrangements, although it lacks the ability to identify balanced chromosome rearrangements, mosaicism, and molar pregnancies [12]. The estimated cost of SNP array and aCGH are € 6000 and € 3600, respectively [40]. A turnover time of 8 and 9 days was reported for SNP array and aCGH, respectively [40]. Both techniques are relatively rapid and less labor-intensive than karyotyping; however, CMA is not available in every practice [46,51].

### 3.4. Comparison of Genetic Tests

We collected information about four different genetic tests for POC analysis, namely karyotyping, QF-PCR, SNP array, and aCGH analysis. Information on cost, time, accessibility, and technical success rate for each test was consolidated to form an overview (Table 2). Karyotyping and SNP array are estimated to be the costliest detection methods, while QF-PCR was by far the most cost-effective. QF-PCR is the most rapid technique with a turnover time of approximately 1–3 days, while karyotyping requires the longest time, at 23 days. Both have a high accessibility compared to SNP array and aCGH. Finally, QF-PCR and SNP array both have a similar technical success rate of 94% and 93%, respectively—meaning they provide interpretable results in the majority of cases. In contrast, conventional karyotyping has a lower technical success rate, ranging between 60 and 90%, depending on tissue quality and culture conditions [28,40,44,45,48,51].

A summary of the types of chromosomal anomalies detectable by each test was compiled (Table 3). SNP array and aCGH detected the least number of types of chromosomal anomalies, not being able to detect mosaicism, molar pregnancies, and balanced structural rearrangements. QF-PCR demonstrated the highest performance, with the exception of its inability to detect tetraploidy and balanced structural rearrangements. Karyotyping is the only technique able to detect balanced structural rearrangements, providing they exceed 5 MB in size. However, it is also the only technique unable to detect MCC [28,30,39,40,47,48,50].

## 4. Autopsy

Fetal autopsy may aid in determining the cause of death in perinatal losses. A study of 226 pregnancy losses reported an overall diagnostic yield of 18.1%, but did not distinguish between miscarriage, preterm birth, and stillbirth. As cases ranged from 15 to 43 weeks of gestation, the applicability of these findings to miscarriages before 16 weeks is limited, as no subgroup analysis by gestational age was performed. Since fetal size influences autopsy success, the diagnostic yield in early miscarriages may be significantly lower [52].

This highlights a significant limitation of performing autopsies in miscarriages: in early miscarriages, the small size of the embryo often makes autopsy procedures nearly impossible. Furthermore, tissue can deteriorate in utero due to maceration, a process that occurs after intrauterine fetal death (IUFD) and involves the breakdown of fetal tissues due to enzymatic autolysis. This series of events causes skin discoloration and the formation of bullae. Moreover, the skin peels and edema accumulates. Maceration makes it increasingly difficult to dissect and anatomically differentiate fetal tissue, making autopsy a suboptimal technique for dissecting early miscarriages [53]. In one study, 75 fetuses collected from miscarriages and stillbirths were examined, and the authors found a total maceration rate of 74.6%, with 32% and 16% being moderately and severely macerated, respectively, posing a significant issue for autopsy [54].

Besides the technical difficulties, the feasibility of performing an autopsy further diminishes due to a worldwide decline in parental consent [55,56,57]. Conventional autopsy is often disliked because of the invasiveness of the procedure, practicalities, or cultural and religious preferences [58]. However, regret about their choice was more common among parents who declined post-mortem examination (14%) compared to those who consented to it (7%), highlighting the potential emotional benefits of post-mortem investigation and the need for less invasive techniques to support decision-making [59].

## 5. Imaging

The desire of parents for a less-invasive alternative to autopsy has led to the development of fetal post-mortem imaging techniques. Different techniques such as microfocus computed tomography (micro-CT), post-mortem ultrasound (PMUS), and ultra-high-field magnetic resonance imaging (UHF-MRI) are increasingly utilized in miscarriage investigation although their application is still primarily confined to research settings [60]. In the next passage, we will explore the use of micro-CT, PMUS, and UHF-MRI for the examination of miscarriages.

### 5.1. Microfocus Computed Tomography

**Figure 1:**
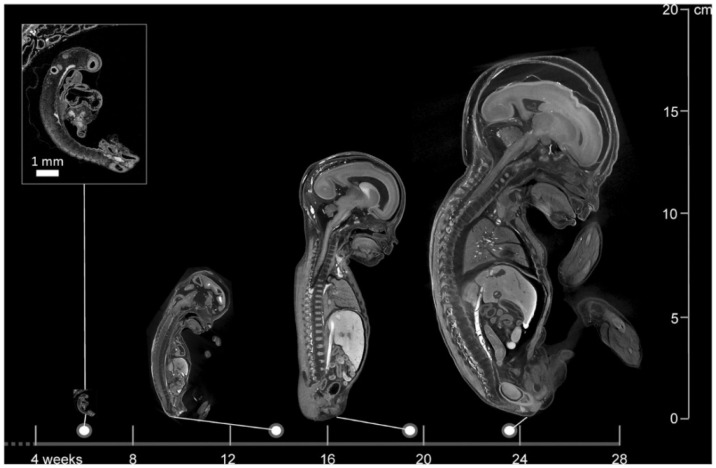
**Images of a 6-week-old embryo and 13-, 20-, and 24-week-old fetuses, created with micro-CT**. This image illustrates the wide applicability in gestational age range of micro-CT. Scale bar represents 1 mm (Dawood et al. 2022 [62], with permission).

Micro-CT is an advanced imaging technique that, like conventional computed tomography (CT), utilizes X-rays to generate detailed images of anatomical structures. Unlike standard CT, micro-CT achieves significantly higher resolution, particularly when imaging smaller areas of interest (Figure 1). This makes it especially suitable for the precise visualization and analysis of fine structural details in small-scale objects, such as POC [61,62].

Micro-CT has already been applied to determine the anatomy of presumed healthy embryos [63,64]. This has enabled the establishment of the 3D Fetal Atlas (unpublished data Dawood et al.), an atlas containing the anatomy and development of fetuses that can serve as reference material [64].

Prior to micro-CT imaging, fetal specimens must undergo contrast enhancement, typically using Lugol’s iodine staining [65]. As tissue shrinkage may occur during the staining process, it is essential to adhere to a standardized protocol to minimize morphological artifacts [66]. Moreover, specimens should be immobilized in agarose gel within a plastic container to prevent movement and ensure optimal image quality during scanning [67,68].

Using autopsy as a comparison, micro-CT appears to give promising results in identifying anatomical anomalies. A study involving 20 fetuses with gestational ages ranging from 11 to 21 weeks reported overall sensitivities of 93.8% and specificities of 100% for detecting congenital defects using micro-CT [69]. Another comprehensive study evaluating 268 micro-CT scans of fetuses between 11 and 24 weeks gestational age documented a sensitivity and specificity of 92.3% and 98%, respectively. These results imply that micro-CT serves as a good alternative to autopsy [70]. Moreover, maceration does not appear to affect micro-CT imaging quality, as high-resolution images were obtained in 95% of cases despite maceration [71]. Furthermore, micro-CT can operate on very small anatomical structures and provides increasing spatial resolution as fetal size decreases, making it a viable alternative where conventional autopsy is unfeasible (Figure 1) [62,70,72].

One functional restriction of micro-CT is that relatively large datafiles, around 50 GB per patient, are produced due to the high resolution of the scans. This requires substantial storage capacity, making it harder to implement [62,72]. Overall, due to the quick scan times, high resolution, and low cost, micro-CT offers distinct advantages alternative to autopsy [73].

### 5.2. Ultra-High-Field Magnetic Resonance Imaging

UHF-MRI refers to MRI with a magnetic field strength greater than 7.0 Tesla, enabling micron-level resolution [67]. Unlike conventional MRI, UHF-MRI is capable of precisely identifying anatomical structures within fetuses below 20 weeks gestational age. However, the resolution of these images is significantly lower than those produced by micro-CT [60,67,68,74]. Furthermore, UHF-MRI is more expensive than micro-CT with an average cost of €400 per specimen, compared to € 200 per specimen for micro-CT. Moreover, the average scan time of UHF-MRI is around three times longer than that of micro-CT [68,74]. Although studies are limited, one investigation evaluated the diagnostic accuracy of 3 T MRI in fetuses exhibiting mild to severe maceration, finding that severe maceration was associated with reduced diagnostic accuracy. It is important to emphasize that these findings were derived using a 3 T MRI system; consequently, the utilization of UHF-MRI may potentially enhance diagnostic accuracy, although UHF-MRI is also known to be more susceptible to artifacts than 3 T MRI [75,76] and has a limited bore diameter.

### 5.3. Post-Mortem Ultrasound

Post-mortem ultrasound (PMUS) is another non-invasive alternative to autopsy. It has the ability to provide data on nearly all body systems [77]. Ultrasound is a widely available and relatively inexpensive technique. The exact cost of PMUS is currently unknown; however, given that conventional prenatal ultrasound is estimated at € 34 per examination, it is reasonable to assume that the cost of PMUS would be comparable [78]. Scanning of a small embryo or fetus provides a challenge, requiring a small but high-resolution probe and submersion of the fetus in water while suspending the covered probe above it [79]. Unlike micro-CT, PMUS does not require staining to improve visibility and thus preserves the original appearance of the fetus.

Maceration makes it increasingly difficult to diagnose anomalies with PMUS, making it the leading contributing factor in non-diagnostic PMUS [80]. Moreover, PMUS has relatively low diagnostic rates of fetuses below 20 weeks gestation, rendering it an ineffective technique to scan miscarriages [81,82]. Despite the downsides, and following the progress in resolution improvement over the years, PMUS could serve as a promising alternative when high-resolution imaging methods are unavailable.

### 5.4. Comparison of Imaging Techniques

**Figure 2:**
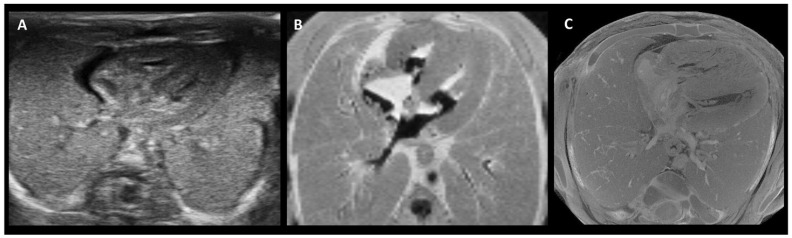
**Side-by-side comparison of PMUS (A), 9.4 T MRI (B), and micro-CT (C) images showing a cross-section of the thorax of a 15-week-old fetus, following termination of pregnancy**. While all three show the general composition of the thorax, micro-CT generated a more detailed image of the structural anatomy. Adapted from [74].

**Figure 3:**
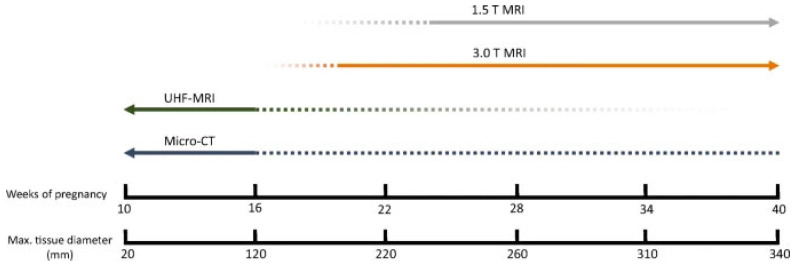
Applicability of different imaging techniques per fetal age. For fetuses younger than 16 weeks of gestation, micro-CT and UHF-MRI appear to be the most suitable modalities [67].

An overview was created of the cost, resolution, scanning time, and the ability to scan despite maceration for the three above-mentioned imaging techniques (Table 4). PMUS offers a relatively simple, accessible, and inexpensive way to scan fetuses. However, the resolution of the images is low and the technique is difficult to apply in miscarriages due to the small size and possible maceration of the fetus [77,79,80,82]. Both UHF-MRI and micro-CT produce relatively high-resolution images, but the resolution of micro-CT is significantly higher and the technique is less time-consuming and estimated to be more cost-effective than UHF-MRI [60,62,67,68,69,74,78,81] (Figure 2). As fetal size increases, micro-CT and UHF-MRI become less effective because of constraints such as the small bore size of UHF-MRI and the longer staining time required for micro-CT. Therefore, for fetuses beyond 20 weeks of gestation, low-field MRI is considered more appropriate (Figure 3) [67].

## 6. Discussion

We aimed to critically examine current strategies for investigating causes of miscarriage through POC analysis, highlighting the strengths and weaknesses of both genetic and morphological techniques. Genetic POC analysis techniques including karyotyping, QF-PCR, SNP array, and aCGH were explored. Karyotyping currently is the historically considered gold standard for genetic analysis of POC in a research setting, although our review shows it might be the least effective technique with a success rate of only 60–90%, mostly due to cell culture failure and MCC. Miscarriage-obtained POC are often more degraded than those derived from termination of pregnancy, resulting in lower quality material and rendering successful cell culture establishment exceedingly difficult, if not nearly impossible. Finally, karyotyping is time-consuming, leading to lengthy waiting times for parents to receive results. With the highest technical success rate, lowest costs, and fastest turnaround time, QF-PCR shows great promise in POC examination. However, QF-PCR is unable to detect tetraploidy and balanced structural rearrangements, occurring in an estimate of 2.8% and 1.6% of the chromosomally abnormal samples, respectively (Table 1). Therefore, in cases where QF-PCR appeared normal, a follow-up technique to identify a broader range of chromosomal anomalies is recommended. Both SNP array and aCGH are suitable for identifying tetraploidy, though only karyotyping can detect balanced structural arrangements, given they are larger than 5 MB. SNP array and aCGH are comparable techniques, offering similar turnaround times and accessibility. Although aCGH has a slightly lower technical success rate, it is more cost-effective than SNP array. Hence, the choice of test largely depends on the availability at the testing facility. Since CMA shows promising results when using FFPE POC samples, it offers the possibility to preserve the fetus and examine it at a later point, which reduces the pressure for a rapid investigation. As the aim of our study was to examine current diagnostic strategies, we did not evaluate new and more advanced molecular techniques, such as whole genome sequencing [83] and concurrent genome-wide haplotyping [84,85]. However, it should be acknowledged that these newer techniques increasingly challenge the traditional designations and methods described in this paper, by enabling more comprehensive and allele-specific characterization of genomic abnormalities. Although these approaches are currently costly and their added clinical value is still being established, they are likely to further refine or redefine existing diagnostic frameworks.

While conventional autopsy is less suitable for analysis of miscarriage tissue and its parental acceptation is declining, post-mortem fetal imaging techniques such as PMUS, UHF-MRI and micro-CT showed promising diagnostic results in early studies. PMUS is the most affordable and easiest implementable technique, which could be a beneficial addition to miscarriage research in medical centers who might not have access to more advanced techniques such as UHF-MRI or micro-CT. However, since PMUS showed the lowest resolution and diagnostic power compared to UHF-MRI and micro-CT, it is not the preferred option. UHF-MRI and micro-CT both generate high resolution images and thereby yield high diagnostic power, although micro-CT significantly excels on both characteristics. It is important to acknowledge the limitation associated with the lengthy staining process required for micro-CT imaging of fetal tissue. Although this step can be time-consuming and needs optimization, the duration is considerably shorter for smaller first-trimester fetuses, which constitute the majority of miscarriage cases. In time-sensitive scenarios, UHF-MRI offers a viable alternative to micro-CT [62]. An additional advantage of employing imaging techniques in miscarriage research is the ability to analyze extra-embryonic structures, such as the placenta and yolk sac, in addition and in connection with the fetus itself. Examining these tissues alongside the embryo can provide more comprehensive insights into the etiology of miscarriage.

No studies have yet combined imaging and genetic analysis of POC to investigate the underlying causes of miscarriage, although their integration could potentially yield a higher diagnostic rate. Current research suggests that understanding the causes of miscarriage may have beneficial effects on parental mental health; however, this hypothesis has not yet been empirically confirmed. Therefore, additional studies are needed to determine the true added value of advanced POC examination techniques in miscarriage research. Despite the substantial societal burden of miscarriage, the use of genetic testing or imaging to improve parental mental health does not currently appear to reduce these costs. At present, such techniques remain relatively expensive due to limited application, though broader implementation in the future may help to lower costs.

In conclusion, miscarriages are common and have a profound impact on the mental health of affected parents. Current international guidelines do not offer routine diagnostic testing to most patients experiencing a miscarriage, leaving over 90% of cases without an identified cause and underscoring a major gap in care. Although genetic analysis and advanced imaging techniques show promising diagnostic potential, we conclude that, consistent with current guidelines, they should not yet be offered in routine clinical care [24]. Instead, they should first be thoroughly investigated within research settings.

A combined approach integrating imaging and genetic analyses of products of conception may ultimately help overcome the limitations of individual techniques and, once validated, could contribute to both improved diagnostic accuracy and better psychological support for parents.

## Figures and Tables

**Table 1:** **Prevalence of different chromosomal anomalies in genetically abnormal POC.** Aneuploidy is by far the most frequent chromosomal abnormality, followed by triploidy, which together make up an average of 81% of the abnormalities. For trisomies, the three most prevalent are included separately. Some data is recorded as not applicable (NA) since certain studies did not report on that type of chromosomal abnormality, or the test employed was unable to detect it. ‘Other’ is used for less common abnormalities such as a monosomy other than X or a double triploidy [30,31,34].

Percentage of Chromosomal Abnormality Types of Abnormal Samples				
	Nagaishi et al. 2004 [31] (N = 423)	Donaghue et al. 2017 [30] (N = 3411)	Popescu-Hobeanu et al. 2022 [34] (N = 230)	Average
**Technique used**	Karyotyping	QF-PCR + aCGH	Karyotyping + QF-PCR	
**Tissue Type**	Chorionic villi	Fetal tissue or chorionic villi	Fetal tissue	
**Trisomy**	61.2%	55.5%	52.6%	56.4%
**Trisomy 16**	15.3%	Most frequent	17.0%	16.2%
**Trisomy 21**	6.6%	Second most frequent	7.4%	7.0%
**Trisomy 22**	6.6%	Third most frequent	6.7%	6.7%
**Monosomy X**	12%	8.3%	17.8%	12.7%
**Triploidy**	13.8%	10.8%	11.9%	12.2%
**Tetraploidy**	2.6%	NA	3.0%	2.8%
**Mosaicism**	3.1%	3.4%	2.2%	2.9%
**Molar pregnancies**	NA	0.2%	NA	0.2%
**Balanced structural rearrangements**	1.0%	NA	2.2%	1.6%
**Unbalanced structural rearrangements**	5.6%	1.6%	5.9%	4.4%
**Other**	0.7%	12.4%	4.4%	5.8%

**Table 2:** **Overview of cost, time, accessibility, and technical success rate of karyotyping, QF-PCR, SNP array, and aCGH.** Karyotyping and SNP array are the costliest, while QF-PCR is the most cost-effective. QF-PCR is the most rapid, with an estimated turnover time of one to three days, while karyotyping is the most time-consuming, requiring around 23 days. However, both are highly accessible. SNP array and QF-PCR perform the best on diagnosing, with a nearly identical technical success rate of 93% and 94%, respectively, while karyotyping achieved a technical success rate of just 60–90% [28,40,44,45,46,48,51].

	Cost	Time	Accessibility	Technical Success Rate
**Karyotyping**	€ 800–6000 [40,42,43]	23 days [12]	High [10]	60–90% [11]
**QF-PCR**	€ 94 [13]	1–3 days [13,14,15,16,17,18,19,20,21,22,23,24,25,26,27,28,29,30,31,32,33,34,35,36,37,38,39,40,41,42,43,44,45,46,47,48,49,50,51,52,53,54,55]	High [13]	94% [14]
**SNP Array**	€ 6012 [12]	8 days [12]	Moderate [15]	93% [12]
**aCGH**	€ 3644 [12]	9 days [12]	Moderate [15]	85% [12]

**Table 3:** **Types of chromosomal anomalies detected by karyotyping, QF-PCR, SNP array, and aCGH.** QF-PCR outperforms the other techniques, being merely incapable of identifying tetraploidy and balanced structural rearrangements. Karyotyping is the only technique able to detect balanced structural rearrangements, given they are >5 MB in size. However, karyotyping is also the sole method unable to detect MCC. SNP array and aCGH identify the least amount of chromosomal abnormalities, unable to detect balanced structural rearrangements, molar pregnancies, and mosaicism [28,30,39,40,47,48,50].

Chromosomal Anomaly	Karyotyping	QF-PCR	SNP Array	aCGH
**Aneuploidy**	+	+	+	+
**Triploidy**	+	+	+	+
**Tetraploidy**	+	−	+	+
**Mosaicism**	−/+	+	−	−
**Molar pregnancies**	+	+	−	−
**Balanced structural rearrangements**	>5 MB	−	−	−
**Unbalanced structural rearrangements**	>5 MB	+	+	+
**Maternal cell contamination (MCC)**	−	+	+	+

**Table 4:** **Comparison of the different imaging techniques of miscarriages.** Post-mortem ultrasound (PMUS) has a short scanning time; however, the resolution is low and PMUS often fails due to maceration. UHF-MRI and micro-CT both have a higher resolution, with micro-CT exceeding the quality of UHF-MRI. UHF-MRI has a longer scanning time and is more expensive than micro-CT. Furthermore, micro-CT is able to generate a high-quality image despite maceration. Limited research has been performed analyzing MRI image quality in spite of maceration.

	PMUS	UHF-MRI	Mirco-CT
**Cost**	€ 34	€ 400	€ 200
**Resolution**	Low	Moderate	High
**Time to scan**	Low	High	Moderate
**Detection despite maceration**	Mostly impossible	Might be possible	Possible

## Data Availability

Not applicable.

## Abbreviations

The following abbreviations are used in this manuscript:

aCGH	array-based Comparative Genomic Hybridization
BMI	Body Mass Index
CMA	Chromosomal Microarray Analysis
CNV	Copy Number Variation
CT	Computed Tomography
ESHRE	European Society of Human Reproduction and Embryology
FFPE	Formalin-Fixed Paraffin-Embedded
FISH	Fluorescence In Situ Hybridization
IUFD	Intrauterine Fetal Death
MCC	Maternal Cell Contamination
MLPA	Multiplex Ligation-Dependent Probe Amplification
MRI	Magnetic Resonance Imaging
NIPT	Non-Invasive Prenatal Testing
OECD	Organization for Economic Cooperation and Development
PMUS	Post-Mortem Ultrasound
POC	Products of Conception
QF-PCR	Quantitative Fluorescence Polymerase Chain Reaction
RPL	Recurrent Pregnancy Loss
SNP	Single Nucleotide Polymorphism
STR	Short Tandem Repeat
UHF-MRI	Ultra-High-Field Magnetic Resonance Imaging

## References

[1] Quenby S., Gallos I.D., Dhillon-Smith R.K., Podesek M., Stephenson M.D., Fisher J., Brosens J.J., Brewin J., Ramhorst R., Lucas E.S. (2021). Miscarriage matters: The epidemiological, physical, psychological, and economic costs of early pregnancy loss. Lancet.

[2] Nederlands Huisartsen Genootschap (2017). Miskraam|NHG-Richtlijnen. https://richtlijnen.nhg.org/standaarden/miskraam.

[3] Kennisinstituut van de Federatie van Medisch Specialisten Miskraam-Richtlijnendatabase. https://richtlijnendatabase.nl/richtlijn/miskraam/startpagina_-_miskraam.html.

[4] Wilcox A.J., Weinberg C.R., O’Connor J.F., Baird D.D., Schlatterer J.P., Canfield R.E., Armstrong E.G., Nisula B.C. (1988). Incidence of early loss of pregnancy. N. Engl. J. Med..

[5] ESHRE Recurrent Pregnancy Loss Guideline Development Group (2022). Recurrent Pregnancy Loss—Guideline of European Society of Human Reproduction and Embryology. https://www.eshre.eu/guidelines-and-legal/guidelines/recurrent-pregnancy-loss.

[6] (2024). Low Fertility in the EU: A Review of Trends and Drivers|Knowledge for Policy. https://knowledge4policy.ec.europa.eu/news/low-fertility-eu-review-trends-drivers_en.

[7] OECD (2024). Declining Fertility Rates Put Prosperity of Future Generations at Risk. https://www.oecd.org/en/about/news/press-releases/2024/06/declining-fertility-rates-put-prosperity-of-future-generations-at-risk.html.

[8] WHO (2023). 1 in 6 People Globally Affected by Infertility. https://www.who.int/news/item/04-04-2023-1-in-6-people-globally-affected-by-infertility.

[9] van den Berg M.M.J., van Maarle M.C., van Wely M., Goddijn M. (2012). Genetics of early miscarriage. Biochim. Biophys. Acta.

[10] Turco M.Y., Moffett A. (2019). Development of the human placenta. Development.

[11] Lucas E.S., Dyer N.P., Murakami K., Lee Y.H., Chan Y.-W., Grimaldi G., Muter J., Brighton P.J., Moore J.D., Patel G. (2016). Loss of Endometrial Plasticity in Recurrent Pregnancy Loss. Stem Cells.

[12] Lucas E.S., Vrljicak P., Muter J., Diniz-da-Costa M.M., Brighton P.J., Kong C.-S., Lipecki J., Fishwick K.J., Odendaal J., Ewington L.J. (2020). Recurrent pregnancy loss is associated with a pro-senescent decidual response during the peri-implantation window. Commun. Biol..

[13] Armstrong B.G., McDonald A.D., Sloan M. (1992). Cigarette, alcohol, and coffee consumption and spontaneous abortion. Am. J. Public Health.

[14] Pandey S., Tyagi R. (2014). Risk factors for miscarriage from a prevention perspective: A nationwide follow-up study. BJOG Int. J. Obstet. Gynaecol..

[15] Woelfer B., Salim R., Banerjee S., Elson J., Regan L., Jurkovic D. (2001). Reproductive outcomes in women with congenital uterine anomalies detected by three-dimensional ultrasound screening. Obstet. Gynecol..

[16] Giakoumelou S., Wheelhouse N., Cuschieri K., Entrican G., Howie S.E.M., Horne A.W. (2016). The role of infection in miscarriage. Hum. Reprod. Update.

[17] Zhang L., Liu W., Hou K., Lin J., Zhou C., Tong X., Wang Z., Wang Y., Jiang Y., Wang Z. (2019). Air pollution-induced missed abortion risk for pregnancies. Nat. Sustain..

[18] Kersting A., Wagner B. (2012). Complicated grief after perinatal loss. Dialogues Clin. Neurosci..

[19] Farren J., Mitchell-Jones N., Verbakel J.Y., Timmerman D., Jalmbrant M., Bourne T. (2018). The psychological impact of early pregnancy loss. Hum. Reprod. Update.

[20] Farren J., Jalmbrant M., Falconieri N., Mitchell-Jones N., Bobdiwala S., Al-Memar M., Tapp S., Van Calster B., Wynants L., Timmerman D. (2020). Posttraumatic stress, anxiety and depression following miscarriage and ectopic pregnancy: A multicenter, prospective, cohort study. Am. J. Obstet. Gynecol..

[21] Dawood Y., de Vries J.M., van Leeuwen E., van Eekelen R., de Bakker B.S., Boelen P.A., Pajkrt E. (2024). Psychological sequelae following second-trimester termination of pregnancy: A longitudinal study. Acta Obstet. Gynecol. Scand..

[22] Bardos J., Hercz D., Friedenthal J., Missmer S.A., Williams Z. (2015). A national survey on public perceptions of miscarriage. Obstet. Gynecol..

[23] American College of Obstetricians and Gynecologists (2002). ACOG practice bulletin. Management of recurrent pregnancy loss. Number 24, February 2001. (Replaces Technical Bulletin Number 212, September 1995). American College of Obstetricians and Gynecologists. Int. J. Gynaecol. Obstet. Off. Organ Int. Fed. Gynaecol. Obstet..

[24] Atik R.B., Christiansen O.B., Elson J., Kolte A.M., Lewis S., Middeldorp S., Mcheik S., Peramo B., Quenby S., The ESHRE Guideline Group on RPL (2023). ESHRE guideline: Recurrent pregnancy loss: An update in 2022. Hum. Reprod. Open.

[25] Kennisinstituut van de Federatie van Medisch Specialisten (2023). Herhaalde Miskraam—Richtlijnendatabase. https://richtlijnendatabase.nl/richtlijn/adaptatietraject_internationale_richtlijn_herhaalde_miskraam/herhaalde_miskraam.html.

[26] Regan L., Rai R., Saravelos S., Li T.C., Royal College of Obstetricians and Gynaecologists (2023). Recurrent MiscarriageGreen-top Guideline No. 17. BJOG Int. J. Obstet. Gynaecol..

[27] DGGG, OEGGG, SGGG (2022). Diagnostik und Therapie von Frauen Mit Wiederholten Spontanaborten.

[28] Gao J., Liu C., Yao F., Hao N., Zhou J., Zhou Q., Zhang L., Liu X., Bian X., Liu J. (2012). Array-based comparative genomic hybridization is more informative than conventional karyotyping and fluorescence in situ hybridization in the analysis of first-trimester spontaneous abortion. Mol. Cytogenet..

[29] Wang Y., Zhou R., Jiang L., Meng L., Tan J., Qiao F., Wang Y., Zhang C., Cheng Q., Jiang Z. (2021). Identification of Chromosomal Abnormalities in Early Pregnancy Loss Using a High-Throughput Ligation-Dependent Probe Amplification-Based Assay. J. Mol. Diagn..

[30] Donaghue C., Davies N., Ahn J.W., Thomas H., Ogilvie C.M., Mann K. (2017). Efficient and cost-effective genetic analysis of products of conception and fetal tissues using a QF-PCR/array CGH strategy; five years of data. Mol. Cytogenet..

[31] Nagaishi M., Yamamoto T., Iinuma K., Shimomura K., Berend S.A., Knops J. (2004). Chromosome abnormalities identified in 347 spontaneous abortions collected in Japan. J. Obstet. Gynaecol. Res..

[32] Fritz B., Hallermann C., Olert J., Fuchs B., Bruns M., Aslan M., Schmidt S., Coerdt W., Müntefering H., Rehder H. (2001). Cytogenetic analyses of culture failures by comparative genomic hybridisation (CGH)–Re-evaluation of chromosome aberration rates in early spontaneous abortions. Eur. J. Hum. Genet..

[33] Mardy A., Wapner R.J. (2016). Confined placental mosaicism and its impact on confirmation of NIPT results. Am. J. Med. Genet. C Semin. Med. Genet..

[34] Popescu-Hobeanu G., Riza A.-L., Streață I., Tudorache Ș., Comănescu A., Tănase F., Drăgușin R.C., Pascu C., Dijmărescu A.L., Cara M.-L. (2022). Cytogenetic Analysis of Sporadic First-Trimester Miscarriage Specimens Using Karyotyping and QF-PCR: A Retrospective Romanian Cohort Study. Genes.

[35] Hardy K., Hardy P.J. (2015). 1st trimester miscarriage: Four decades of study. Transl. Pediatr..

[36] Goddijn M., Leschot N.J. (2000). Genetic aspects of miscarriage. Baillieres Best Pract. Res. Clin. Obstet. Gynaecol..

[37] De Braekeleer M., Dao T.N. (1990). Cytogenetic studies in couples experiencing repeated pregnancy losses. Hum. Reprod..

[38] Yusuf R.Z., Naeem R. (2004). Cytogenetic abnormalities in products of conception: A relationship revisited. Am. J. Reprod. Immunol..

[39] McQueen D.B., Lathi R.B. (2019). Miscarriage chromosome testing: Indications, benefits and methodologies. Semin. Perinatol..

[40] Shah M.S., Cinnioglu C., Maisenbacher M., Comstock I., Kort J., Lathi R.B. (2017). Comparison of cytogenetics and molecular karyotyping for chromosome testing of miscarriage specimens. Fertil. Steril..

[41] Bell K.A., Van Deerlin P.G., Haddad B.R., Feinberg R.F. (1999). Cytogenetic diagnosis of “normal 46,XX” karyotypes in spontaneous abortions frequently may be misleading. Fertil. Steril..

[42] Qian G., Cai L., Yao H., Dong X. (2023). Chromosome microarray analysis combined with karyotype analysis is a powerful tool for the detection in pregnant women with high-risk indicators. BMC Pregnancy Childbirth.

[43] (2025). Tarieven Medisch Specialistische Zorg per 1 Januari 2025. CZ. https://czdirect.cz.nl/-/media/files/czdirect/actueel/voorwaarden/gemiddeld-ongewogen-gecontracteerde-tarieven-msz.pdf?sc_lang=nl-NL&hash=76E4584F4A8A8C61B5B5080A9394EA93.

[44] Donaghue C., Mann K., Docherty Z., Mazzaschi R., Fear C., Ogilvie C. (2010). Combined QF-PCR and MLPA molecular analysis of miscarriage products: An efficient and robust alternative to karyotype analysis. Prenat. Diagn..

[45] Cottino L., Sahibdeen V., Mudau M., Lekgate N., Krause A. (2022). QF-PCR: A valuable first-line prenatal and postnatal test for common aneuploidies in South Africa. J. Community Genet..

[46] Badenas C., Rodríguez-Revenga L., Morales C., Mediano C., Plaja A., Pérez-Iribarne M.M., Soler A., Clusellas N., Borrell A., Sánchez M.Á. (2010). Assessment of QF-PCR as the First Approach in Prenatal Diagnosis. J. Mol. Diagn..

[47] Mann K., Hamilton S., Evans J., Sibbring J., Dore J. (2018). Best Practice Guidelines for use of Quantitative Fluorescence-PCR for the detection of aneuploidy. Assoc. Clin. Genom. Sci..

[48] Diego-Alvarez D., Garcia-Hoyos M., Trujillo M.J., Gonzalez-Gonzalez C., de Alba M.R., Ayuso C., Ramos-Corrales C., Lorda-Sanchez I. (2005). Application of quantitative fluorescent PCR with short tandem repeat markers to the study of aneuploidies in spontaneous miscarriages. Hum. Reprod..

[49] Stravopoulus D.J. (2023). Principles of Clinical Cytogenetics and Genome Analysis. Thompson & Thompson Genetics and Genomics in Medicine.

[50] Sahoo T., Dzidic N., Strecker M.N., Commander S., Travis M.K., Doherty C., Tyson R.W., Mendoza A.E., Stephenson M., Dise C.A. (2017). Comprehensive genetic analysis of pregnancy loss by chromosomal microarrays: Outcomes, benefits, and challenges. Genet. Med. Off. J. Am. Coll. Med. Genet..

[51] Dahdouh E.M., Kutteh W.H. (2021). Genetic testing of products of conception in recurrent pregnancy loss evaluation. Reprod. Biomed. Online.

[52] Neşe N., Bülbül Y. (2018). Diagnostic value of perinatal autopsies: Analysis of 486 cases. J. Perinat. Med..

[53] Yhee Khong T., Malcomson R.D.G. (2022). Macerated stillbirth. Keeling’s Fetal and Neonatal Pathology.

[54] Montaldo P., Addison S., Oliveira V., Lally P.J., Taylor A.M., Sebire N.J., Thayyil S., Arthurs O.J. (2016). Quantification of maceration changes using post mortem MRI in fetuses. BMC Med. Imaging.

[55] Stock S.J., Goldsmith L., Evans M.J., Laing I.A. (2010). Interventions to improve rates of post-mortem examination after stillbirth. Eur. J. Obstet. Gynecol. Reprod. Biol..

[56] Kock K.F., Vestergaard V., Hardt-Madsen M., Garne E. (2003). Declining autopsy rates in stillbirths and infant deaths: Results from Funen County, Denmark, 1986–96. J. Matern.-Fetal Neonatal Med..

[57] Lewis C., Riddington M., Hill M., Arthurs O.J., Hutchinson J.C., Chitty L.S., Bevan C., Fisher J., Ward J., Sebire N.J. (2019). Availability of less invasive prenatal, perinatal and paediatric autopsy will improve uptake rates: A mixed-methods study with bereaved parents. BJOG Int. J. Obstet. Gynaecol..

[58] Lewis C., Hill M., Arthurs O.J., Hutchinson C., Chitty L.S., Sebire N.J. (2018). Factors affecting uptake of postmortem examination in the prenatal, perinatal and paediatric setting. BJOG Int. J. Obstet. Gynaecol..

[59] Rankin J., Wright C., Lind T. (2002). Cross sectional survey of parents’ experience and views of the postmortem examination. BMJ.

[60] Simcock I.C., Lamouroux A., Sebire N.J., Shelmerdine S.C., Arthurs O.J. (2023). Less-invasive autopsy for early pregnancy loss. Prenat. Diagn..

[61] Orhan K., Orhan K. (2020). Introduction to Micro-CT Imaging. Micro-Computed Tomography (Micro-CT) in Medicine and Engineering.

[62] Dawood Y., Buijtendijk M.F., Shah H., Smit J.A., Jacobs K., Hagoort J., Oostra R.-J., Bourne T., van den Hoff M.J.B., de Bakker B.S. (2022). Imaging fetal anatomy. Semin. Cell Dev. Biol..

[63] Shelmerdine S.C., Hutchinson J.C., Kang X., Suich J.D., Ashworth M., Cannie M.M., Segers V., Sebire N.J., Jani J.C., Arthurs O.J. (2018). Novel usage of microfocus computed tomography (micro-CT) for visualisation of human embryonic development-Implications for future non-invasive post-mortem investigation. Prenat. Diagn..

[64] de Bakker B.S., de Jong K.H., Hagoort J., de Bree K., Besselink C.T., de Kanter F.E.C., Veldhuis T., Bais B., Schildmeijer R., Ruijter J.M. (2016). An interactive three-dimensional digital atlas and quantitative database of human development. Science.

[65] Docter D., Dawood Y., Jacobs K., Hagoort J., Oostra R.-J., van den Hoff M.J.B., Arthurs O.J., de Bakker B.S. (2023). Microfocus computed tomography for fetal postmortem imaging: An overview. Pediatr. Radiol..

[66] Dawood Y., Hagoort J., Siadari B.A., Ruijter J.M., Gunst Q.D., Lobe N.H.J., Strijkers G.J., de Bakker B.S., van den Hoff M.J.B. (2021). Reducing soft-tissue shrinkage artefacts caused by staining with Lugol’s solution. Sci. Rep..

[67] Dawood Y., Strijkers G.J., Limpens J., Oostra R.J., de Bakker B.S. (2020). Novel imaging techniques to study postmortem human fetal anatomy: A systematic review on microfocus-CT and ultra-high-field MRI. Eur. Radiol..

[68] Dawood Y., Honhoff C., van der Post A.S., Roosendaal S.D., Coolen B.F., Strijkers G.J., Pajkrt E., de Bakker B.S. (2022). Comparison of postmortem whole-body contrast-enhanced microfocus computed tomography and high-field magnetic resonance imaging of human fetuses. Ultrasound Obstet. Gynecol..

[69] Hutchinson J.C., Kang X., Shelmerdine S.C., Segers V., Lombardi C.M., Cannie M.M., Sebire N.J., Jani J.C., Arthurs O.J. (2018). Postmortem microfocus computed tomography for early gestation fetuses: A validation study against conventional autopsy. Am. J. Obstet. Gynecol..

[70] Shelmerdine S.C., Simcock I.C., Hutchinson J.C., Guy A., Ashworth M.T., Sebire N.J., Arthurs O.J. (2021). Postmortem microfocus computed tomography for noninvasive autopsies: Experience in >250 human fetuses. Am. J. Obstet. Gynecol..

[71] Simcock I.C., Shelmerdine S.C., Langan D., Anna G., Sebire N.J., Arthurs O.J. (2021). Micro-CT yields high image quality in human fetal post-mortem imaging despite maceration. BMC Med. Imaging.

[72] Simcock I.C., Shelmerdine S.C., Hutchinson J.C., Sebire N.J., Arthurs O.J. (2021). Human fetal whole-body postmortem microfocus computed tomographic imaging. Nat. Protoc..

[73] Sandaite I., Lombardi C., Cook A.C., Fabietti I., Deprest J., Boito S. (2020). Micro-computed tomography of isolated fetal hearts following termination of pregnancy: A feasibility study at 8 to 12 weeks’ gestation. Prenat. Diagn..

[74] Vilar P.I., Jani J.C., Cannie M.M., Shelmerdine S.C., Lecomte S., Verhoye M., Hutchinson C.J., Arthurs O.J., Carlin A., Kang X. (2024). Postmortem imaging of fetuses at early gestations: A comparison of microfocus computed tomography with postmortem magnetic resonance at 9.4 T and postmortem ultrasound. Prenat. Diagn..

[75] Ulm B., Dovjak G.O., Scharrer A., Muin D.A., Zimpfer D., Prayer D., Weber M., Berger-Kulemann V. (2021). Diagnostic quality of 3Tesla postmortem magnetic resonance imaging in fetuses with and without congenital heart disease. Am. J. Obstet. Gynecol..

[76] Cramer J., Ikuta I., Zhou Y. (2024). How to Implement Clinical 7T MRI—Practical Considerations and Experience with Ultra-High-Field MRI. Bioengineering.

[77] Shelmerdine S.C., Sebire N.J., Arthurs O.J. (2019). Perinatal post-mortem ultrasound (PMUS): Radiological-pathological correlation. Insights Imaging.

[78] Hendrix M.J., Evers S.M., Basten M.C., Nijhuis J.G., Severens J.L. (2009). Cost Analysis of the Dutch Obstetric System: Low-risk nulliparous women preferring home or short-stay hospital birth—A prospective non-randomised controlled study. BMC Health Serv. Res..

[79] Shelmerdine S.C., Sebire N.J., Arthurs O.J. (2019). Perinatal post mortem ultrasound (PMUS): A practical approach. Insights Imaging.

[80] Shelmerdine S.C., Langan D., Mandalia U., Sebire N.J., Arthurs O.J. (2020). Maceration determines diagnostic yield of fetal and neonatal whole body post-mortem ultrasound. Prenat. Diagn..

[81] Shelmerdine S.C., Sebire N.J., Arthurs O.J. (2021). Diagnostic accuracy of postmortem ultrasound vs postmortem 1.5-T MRI for non-invasive perinatal autopsy. Ultrasound Obstet. Gynecol..

[82] Kang X., Sanchez T.C., Arthurs O.J., Bevilacqua E., Cannie M.M., Segers V., Lecomte S., Sebire N.J., Jani J.C. (2019). Postmortem fetal imaging: Prospective blinded comparison of two-dimensional ultrasound with magnetic resonance imaging. Ultrasound Obstet. Gynecol..

[83] Arnadottir G.A., Jonsson H., Hartwig T.S., Gruhn J.R., Møller P.L., Gylfason A., Westergaard D., Chan A.C.-H., Oddsson A., Stefansdottir L. (2025). Sequence diversity lost in early pregnancy. Nature.

[84] Esteki M.Z., Dimitriadou E., Mateiu L., Melotte C., Van der Aa N., Kumar P., Das R., Theunis K., Cheng J., Legius E. (2015). Concurrent whole-genome haplotyping and copy-number profiling of single cells. Am. J. Hum. Genet..

[85] Essers R., Lebedev I.N., Kurg A., Fonova E.A., Stevens S.J.C., Koeck R.M., von Rango U., Brandts L., Deligiannis S.P., Nikitina T.V. (2023). Prevalence of chromosomal alterations in first-trimester spontaneous pregnancy loss. Nat. Med..

